# State Medical Society

**Published:** 1873-02

**Authors:** 


STATE MEDICAL SOCIETY.—We are able through the kindness of our friend Dr. F. C. Curtis, of Albany, to present to our readers a full report of the proceedings of the State Medical Society at Albany. It will be seen to have been a meeting of considerable interest.

